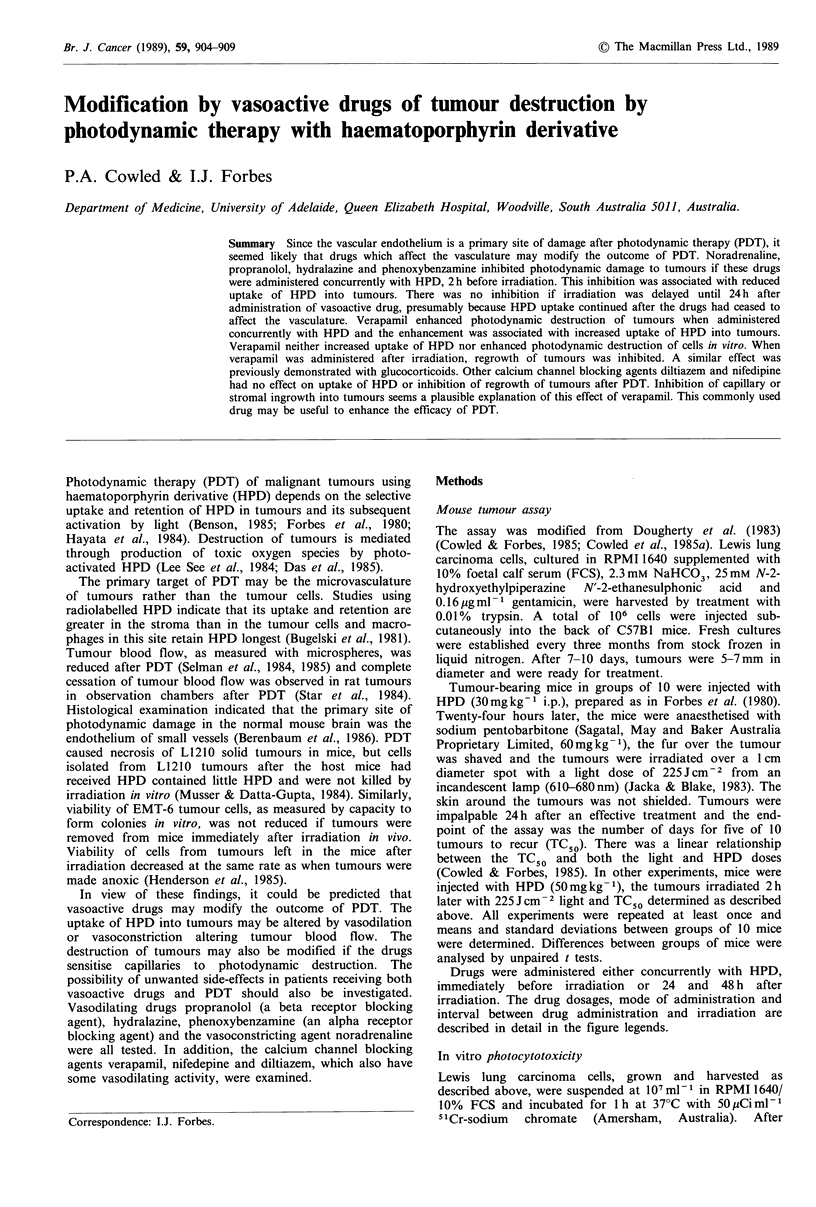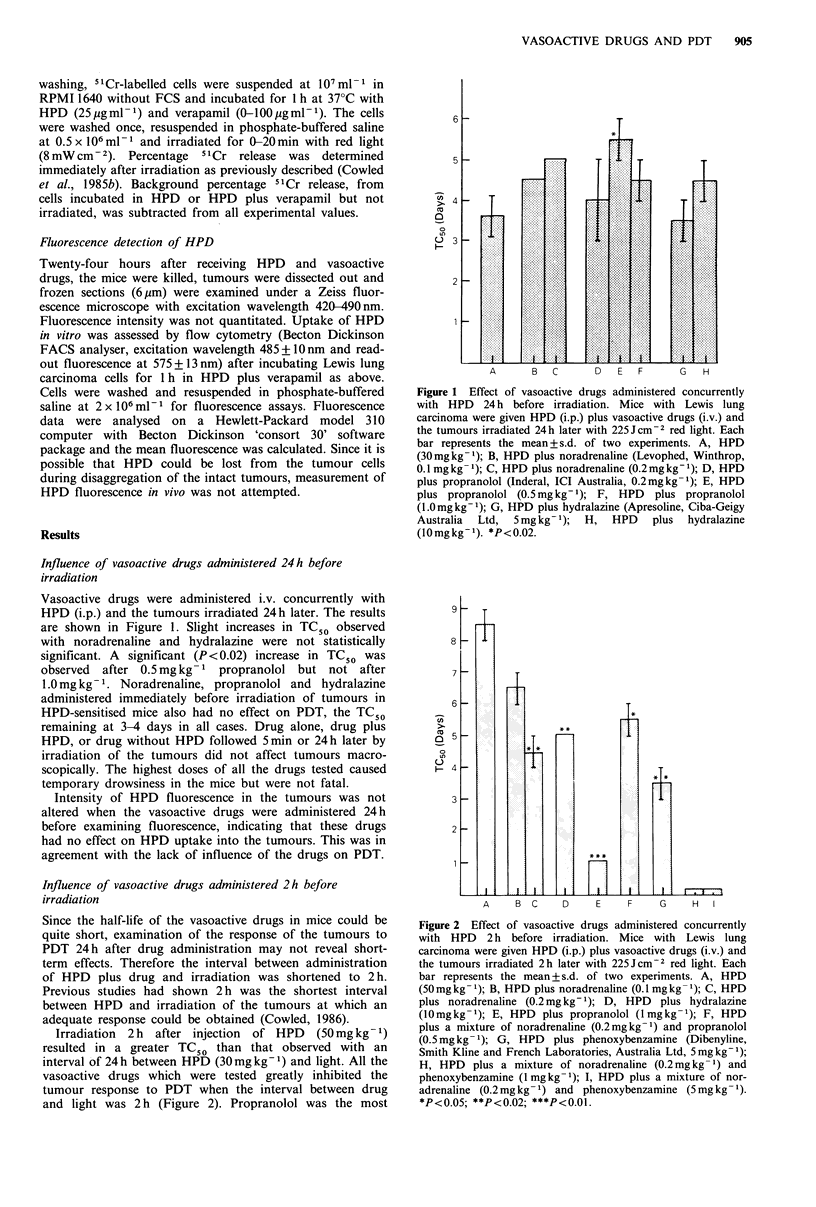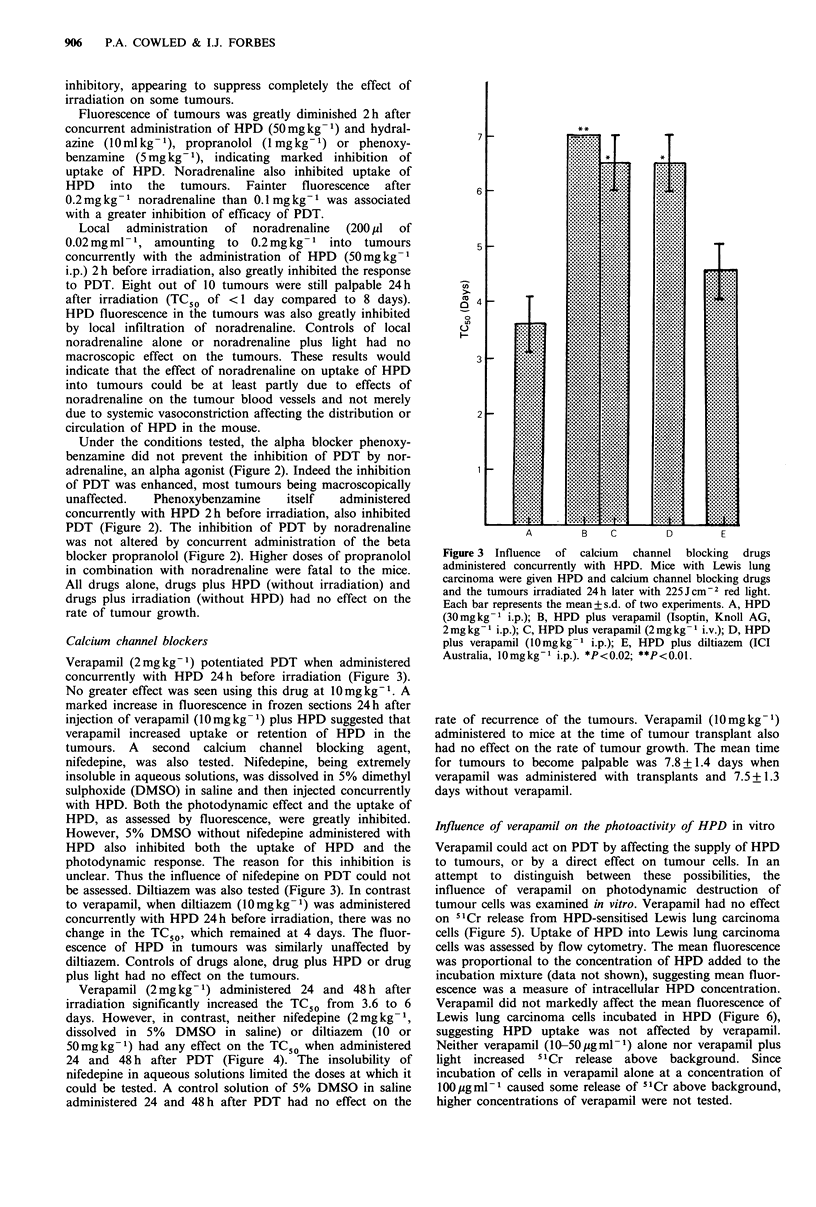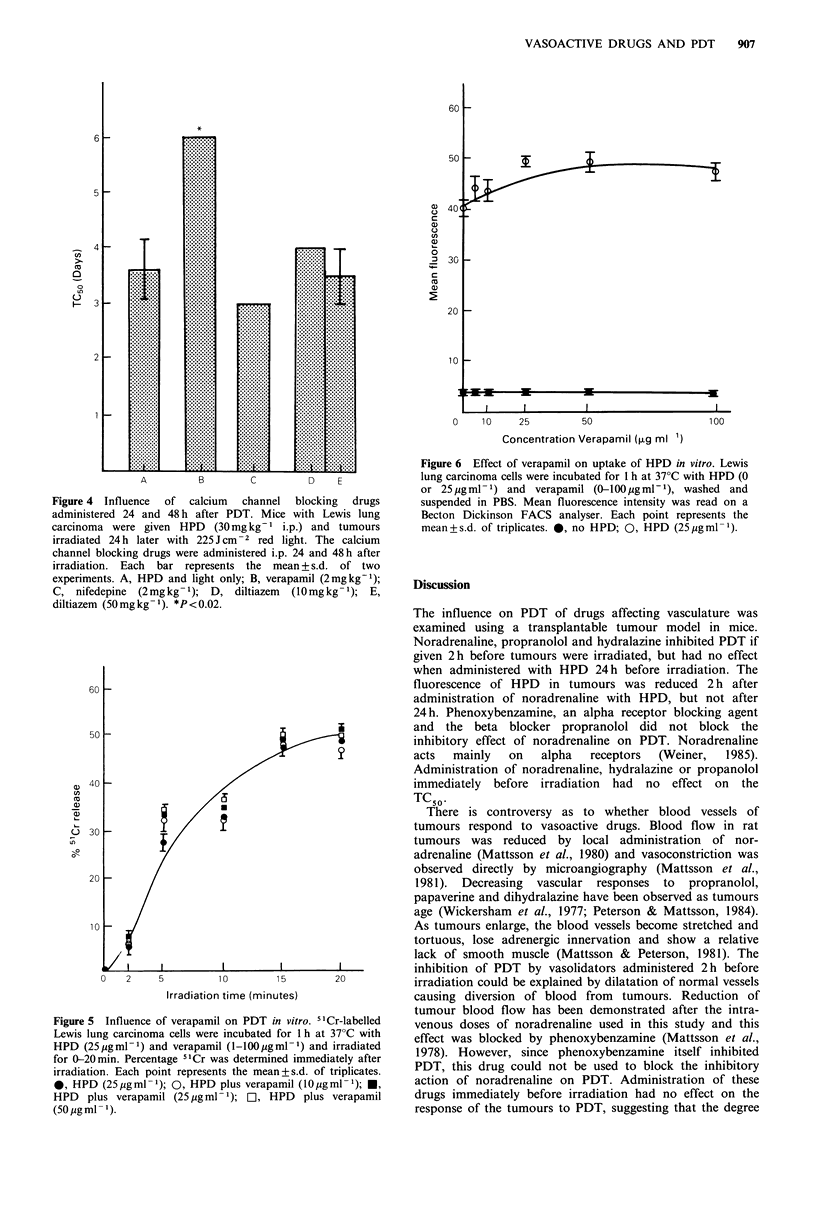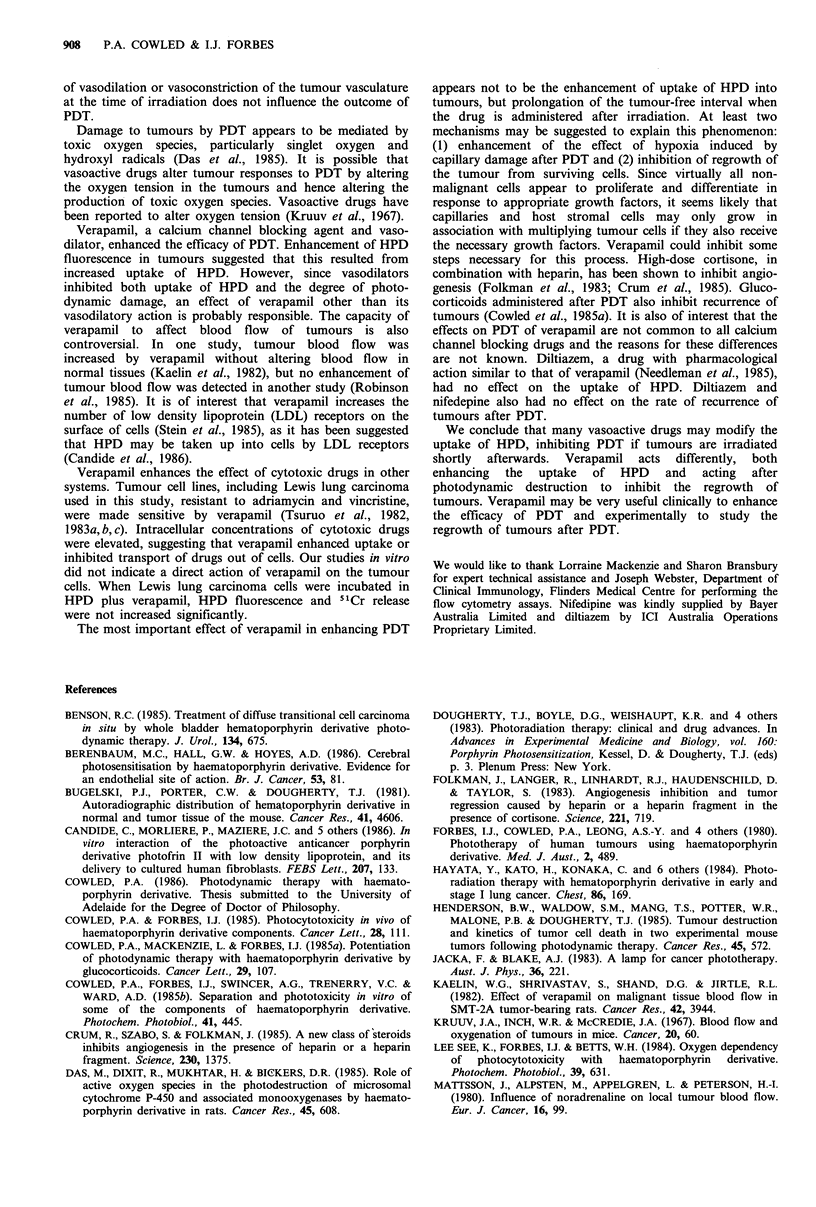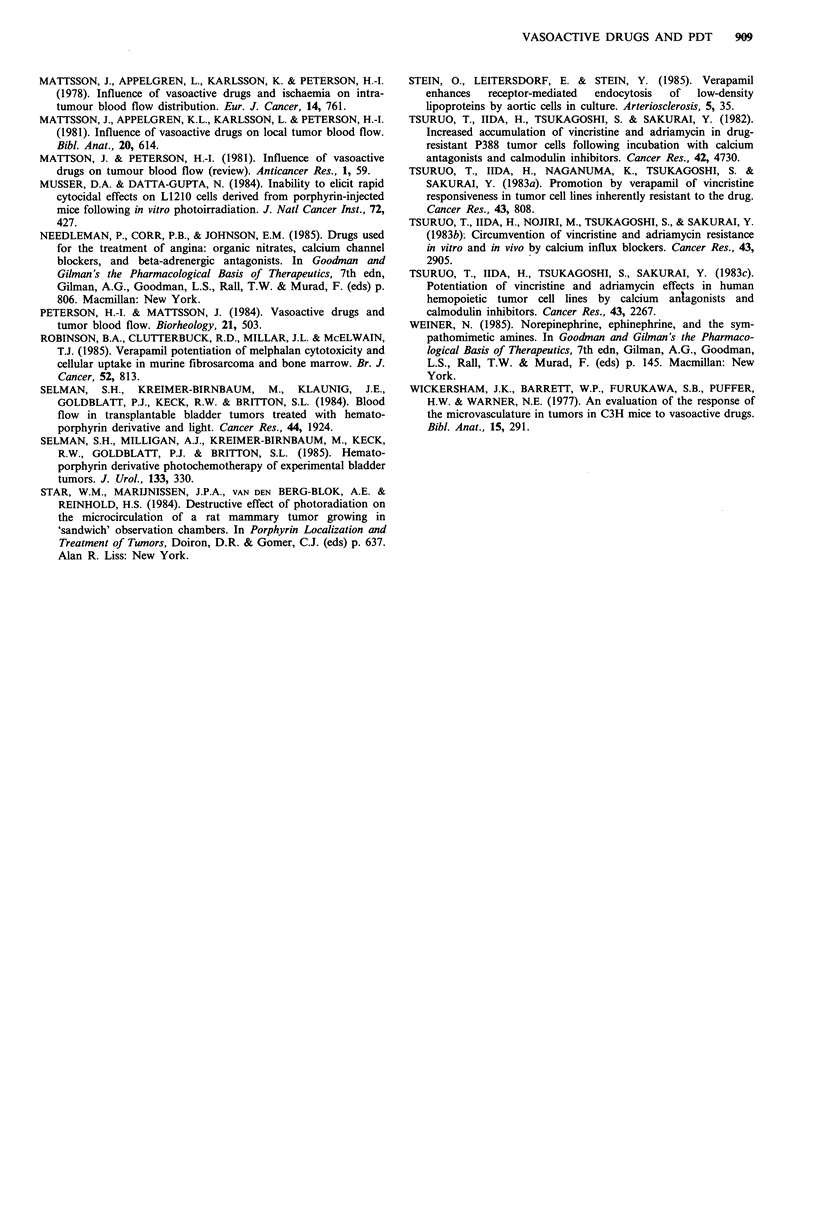# Modification by vasoactive drugs of tumour destruction by photodynamic therapy with haematoporphyrin derivative.

**DOI:** 10.1038/bjc.1989.191

**Published:** 1989-06

**Authors:** P. A. Cowled, I. J. Forbes

**Affiliations:** Department of Medicine, University of Adelaide, Queen Elizabeth Hospital, Woodville, South Australia.

## Abstract

Since the vascular endothelium is a primary site of damage after photodynamic therapy (PDT), it seemed likely that drugs which affect the vasculature may modify the outcome of PDT. Noradrenaline, propranolol, hydralazine and phenoxybenzamine inhibited photodynamic damage to tumours if these drugs were administered concurrently with HPD, 2 h before irradiation. This inhibition was associated with reduced uptake of HPD into tumours. There was no inhibition if irradiation was delayed until 24 h after administration of vasoactive drug, presumably because HPD uptake continued after the drugs had ceased to affect the vasculature. Verapamil enhanced photodynamic destruction of tumours when administered concurrently with HPD and the enhancement was associated with increased uptake of HPD into tumours. Verapamil neither increased uptake of HPD nor enhanced photodynamic destruction of cells in vitro. When verapamil was administered after irradiation, regrowth of tumours was inhibited. A similar effect was previously demonstrated with glucocorticoids. Other calcium channel blocking agents diltiazem and nifedipine had no effect on uptake of HPD or inhibition of regrowth of tumours after PDT. Inhibition of capillary or stromal ingrowth into tumours seems a plausible explanation of this effect of verapamil. This commonly used drug may be useful to enhance the efficacy of PDT.


*Br. J. Cancer* (1989), **59**, 904–909 © The Macmillan Press Ltd., 1989

### Modification by vasoactive drugs of tumour destruction by photodynamic therapy with haematoporphyrin derivative

P.A. Cowled & I.J. Forbes

*Department of Medicine, University of Adelaide, Queen Elizabeth Hospital, Woodville, South Australia 5011, Australia.*

**Summary** Since the vascular endothelium is a primary site of damage after photodynamic therapy (PDT), it seemed likely that drugs which affect the vasculature may modify the outcome of PDT. Noradrenaline, propranolol, hydralazine and phenoxybenzamine inhibited photodynamic damage to tumours if these drugs were administered concurrently with HPD, 2 h before irradiation. This inhibition was associated with reduced uptake of HPD into tumours. There was no inhibition if irradiation was delayed until 24 h after administration of vasoactive drug, presumably because HPD uptake continued after the drugs had ceased to affect the vasculature. Verapamil enhanced photodynamic destruction of tumours when administered concurrently with HPD and the enhancement was associated with increased uptake of HPD into tumours. Verapamil neither increased uptake of HPD nor enhanced photodynamic destruction of cells *in vitro*. When verapamil was administered after irradiation, regrowth of tumours was inhibited. A similar effect was previously demonstrated with glucocorticoids. Other calcium channel blocking agents diltiazem and nifedipine had no effect on uptake of HPD or inhibition of regrowth of tumours after PDT. Inhibition of capillary or stromal ingrowth into tumours seems a plausible explanation of this effect of verapamil. This commonly used drug may be useful to enhance the efficacy of PDT.

Photodynamic therapy (PDT) of malignant tumours using haematoporphyrin derivative (HPD) depends on the selective uptake and retention of HPD in tumours and its subsequent activation by light (Benson, 1985; Forbes *et al.*, 1980; Hayata *et al.*, 1984). Destruction of tumours is mediated through production of toxic oxygen species by photo-activated HPD (Lee See *et al.*, 1984; Das *et al.*, 1985).

The primary target of PDT may be the microvasculature of tumours rather than the tumour cells. Studies using radiolabelled HPD indicate that its uptake and retention are greater in the stroma than in the tumour cells and macrophages in this site retain HPD longest (Bugelski *et al.*, 1981). Tumour blood flow, as measured with microspheres, was reduced after PDT (Selman *et al.*, 1984, 1985) and complete cessation of tumour blood flow was observed in rat tumours in observation chambers after PDT (Star *et al.*, 1984). Histological examination indicated that the primary site of photodynamic damage in the normal mouse brain was the endothelium of small vessels (Berenbaum *et al.*, 1986). PDT caused necrosis of L1210 solid tumours in mice, but cells isolated from L1210 tumours after the host mice had received HPD contained little HPD and were not killed by irradiation *in vitro* (Musser & Datta-Gupta, 1984). Similarly, viability of EMT-6 tumour cells, as measured by capacity to form colonies *in vitro*, was not reduced if tumours were removed from mice immediately after irradiation *in vivo*. Viability of cells from tumours left in the mice after irradiation decreased at the same rate as when tumours were made anoxic (Henderson *et al.*, 1985).

In view of these findings, it could be predicted that vasoactive drugs may modify the outcome of PDT. The uptake of HPD into tumours may be altered by vasodilation or vasoconstriction altering tumour blood flow. The destruction of tumours may also be modified if the drugs sensitise capillaries to photodynamic destruction. The possibility of unwanted side-effects in patients receiving both vasoactive drugs and PDT should also be investigated. Vasodilating drugs propranolol (a beta receptor blocking agent), hydralazine, phenoxybenzamine (an alpha receptor blocking agent) and the vasoconstricting agent noradrenaline were all tested. In addition, the calcium channel blocking agents verapamil, nifedepine and diltiazem, which also have some vasodilating activity, were examined.

Correspondence: I.J. Forbes.

### Methods

### *Mouse tumour assay*

The assay was modified from Dougherty *et al.* (1983) (Cowled & Forbes, 1985; Cowled *et al.*, 1985*a*). Lewis lung carcinoma cells, cultured in RPMI 1640 supplemented with 10% foetal calf serum (FCS), 2.3 mM $NaHCO_3$, 25 mM *N*-2-hydroxyethylpiperazine *N*′-2-ethanesulphonic acid and 0.16 $\mu g\,ml^{-1}$ gentamicin, were harvested by treatment with 0.01% trypsin. A total of $10^6$ cells were injected subcutaneously into the back of C57B1 mice. Fresh cultures were established every three months from stock frozen in liquid nitrogen. After 7–10 days, tumours were 5–7 mm in diameter and were ready for treatment.

Tumour-bearing mice in groups of 10 were injected with HPD (30 $mg\,kg^{-1}$ i.p.), prepared as in Forbes *et al.* (1980). Twenty-four hours later, the mice were anaesthetised with sodium pentobarbitone (Sagatal, May and Baker Australia Proprietary Limited, 60 $mg\,kg^{-1}$), the fur over the tumour was shaved and the tumours were irradiated over a 1 cm diameter spot with a light dose of 225 $J\,cm^{-2}$ from an incandescent lamp (610–680 nm) (Jacka & Blake, 1983). The skin around the tumours was not shielded. Tumours were impalpable 24 h after an effective treatment and the end-point of the assay was the number of days for five of 10 tumours to recur ($TC_{50}$). There was a linear relationship between the $TC_{50}$ and both the light and HPD doses (Cowled & Forbes, 1985). In other experiments, mice were injected with HPD (50 $mg\,kg^{-1}$), the tumours irradiated 2 h later with 225 $J\,cm^{-2}$ light and $TC_{50}$ determined as described above. All experiments were repeated at least once and means and standard deviations between groups of 10 mice were determined. Differences between groups of mice were analysed by unpaired *t* tests.

Drugs were administered either concurrently with HPD, immediately before irradiation or 24 and 48 h after irradiation. The drug dosages, mode of administration and interval between drug administration and irradiation are described in detail in the figure legends.

### In vitro *photocytotoxicity*

Lewis lung carcinoma cells, grown and harvested as described above, were suspended at $10^7\,ml^{-1}$ in RPMI 1640/10% FCS and incubated for 1 h at 37°C with 50 $\mu Ci\,ml^{-1}$ $^{51}Cr$-sodium chromate (Amersham, Australia). After washing, $^{51}Cr$-labelled cells were suspended at $10^7 ml^{-1}$ in RPMI 1640 without FCS and incubated for 1 h at 37°C with HPD ($25 \mu g ml^{-1}$) and verapamil ($0-100 \mu g ml^{-1}$). The cells were washed once, resuspended in phosphate-buffered saline at $0.5 \times 10^6 ml^{-1}$ and irradiated for 0–20 min with red light ($8 mW cm^{-2}$). Percentage $^{51}Cr$ release was determined immediately after irradiation as previously described (Cowled *et al.*, 1985*b*). Background percentage $^{51}Cr$ release, from cells incubated in HPD or HPD plus verapamil but not irradiated, was subtracted from all experimental values.

*Fluorescence detection of HPD*

Twenty-four hours after receiving HPD and vasoactive drugs, the mice were killed, tumours were dissected out and frozen sections ($6 \mu m$) were examined under a Zeiss fluorescence microscope with excitation wavelength 420–490 nm. Fluorescence intensity was not quantitated. Uptake of HPD *in vitro* was assessed by flow cytometry (Becton Dickinson FACS analyser, excitation wavelength $485 \pm 10$ nm and readout fluorescence at $575 \pm 13$ nm) after incubating Lewis lung carcinoma cells for 1 h in HPD plus verapamil as above. Cells were washed and resuspended in phosphate-buffered saline at $2 \times 10^6 ml^{-1}$ for fluorescence assays. Fluorescence data were analysed on a Hewlett-Packard model 310 computer with Becton Dickinson 'consort 30' software package and the mean fluorescence was calculated. Since it is possible that HPD could be lost from the tumour cells during disaggregation of the intact tumours, measurement of HPD fluorescence *in vivo* was not attempted.

### Results

*Influence of vasoactive drugs administered 24 h before irradiation*

Vasoactive drugs were administered i.v. concurrently with HPD (i.p.) and the tumours irradiated 24 h later. The results are shown in Figure 1. Slight increases in $TC_{50}$ observed with noradrenaline and hydralazine were not statistically significant. A significant ($P < 0.02$) increase in $TC_{50}$ was observed after $0.5 mg kg^{-1}$ propranolol but not after $1.0 mg kg^{-1}$. Noradrenaline, propranolol and hydralazine administered immediately before irradiation of tumours in HPD-sensitised mice also had no effect on PDT, the $TC_{50}$ remaining at 3–4 days in all cases. Drug alone, drug plus HPD, or drug without HPD followed 5 min or 24 h later by irradiation of the tumours did not affect tumours macroscopically. The highest doses of all the drugs tested caused temporary drowsiness in the mice but were not fatal.

Intensity of HPD fluorescence in the tumours was not altered when the vasoactive drugs were administered 24 h before examining fluorescence, indicating that these drugs had no effect on HPD uptake into the tumours. This was in agreement with the lack of influence of the drugs on PDT.

*Influence of vasoactive drugs administered 2 h before irradiation*

Since the half-life of the vasoactive drugs in mice could be quite short, examination of the response of the tumours to PDT 24 h after drug administration may not reveal short-term effects. Therefore the interval between administration of HPD plus drug and irradiation was shortened to 2 h. Previous studies had shown 2 h was the shortest interval between HPD and irradiation of the tumours at which an adequate response could be obtained (Cowled, 1986).

Irradiation 2 h after injection of HPD ($50 mg kg^{-1}$) resulted in a greater $TC_{50}$ than that observed with an interval of 24 h between HPD ($30 mg kg^{-1}$) and light. All the vasoactive drugs which were tested greatly inhibited the tumour response to PDT when the interval between drug and light was 2 h (Figure 2). Propranolol was the most

**Figure 1** Effect of vasoactive drugs administered concurrently with HPD 24 h before irradiation. Mice with Lewis lung carcinoma were given HPD (i.p.) plus vasoactive drugs (i.v.) and the tumours irradiated 24 h later with $225 J cm^{-2}$ red light. Each bar represents the mean $\pm$ s.d. of two experiments. A, HPD ($30 mg kg^{-1}$); B, HPD plus noradrenaline (Levophed, Winthrop, $0.1 mg kg^{-1}$); C, HPD plus noradrenaline ($0.2 mg kg^{-1}$); D, HPD plus propranolol (Inderal, ICI Australia, $0.2 mg kg^{-1}$); E, HPD plus propranolol ($0.5 mg kg^{-1}$); F, HPD plus propranolol ($1.0 mg kg^{-1}$); G, HPD plus hydralazine (Apresoline, Ciba-Geigy Australia Ltd, $5 mg kg^{-1}$); H, HPD plus hydralazine ($10 mg kg^{-1}$). $^*P < 0.02$.

**Figure 2** Effect of vasoactive drugs administered concurrently with HPD 2 h before irradiation. Mice with Lewis lung carcinoma were given HPD (i.p.) plus vasoactive drugs (i.v.) and the tumours irradiated 2 h later with $225 J cm^{-2}$ red light. Each bar represents the mean $\pm$ s.d. of two experiments. A, HPD ($50 mg kg^{-1}$); B, HPD plus noradrenaline ($0.1 mg kg^{-1}$); C, HPD plus noradrenaline ($0.2 mg kg^{-1}$); D, HPD plus hydralazine ($10 mg kg^{-1}$); E, HPD plus propranolol ($1 mg kg^{-1}$); F, HPD plus a mixture of noradrenaline ($0.2 mg kg^{-1}$) and propranolol ($0.5 mg kg^{-1}$); G, HPD plus phenoxybenzamine (Dibenyline, Smith Kline and French Laboratories, Australia Ltd, $5 mg kg^{-1}$); H, HPD plus a mixture of noradrenaline ($0.2 mg kg^{-1}$) and phenoxybenzamine ($1 mg kg^{-1}$); I, HPD plus a mixture of noradrenaline ($0.2 mg kg^{-1}$) and phenoxybenzamine ($5 mg kg^{-1}$). $^*P < 0.05$; $^{**}P < 0.02$; $^{***}P < 0.01$.

inhibitory, appearing to suppress completely the effect of irradiation on some tumours.

Fluorescence of tumours was greatly diminished 2h after concurrent administration of HPD ($50\,mg\,kg^{-1}$) and hydralazine ($10\,ml\,kg^{-1}$), propranolol ($1\,mg\,kg^{-1}$) or phenoxybenzamine ($5\,mg\,kg^{-1}$), indicating marked inhibition of uptake of HPD. Noradrenaline also inhibited uptake of HPD into the tumours. Fainter fluorescence after $0.2\,mg\,kg^{-1}$ noradrenaline than $0.1\,mg\,kg^{-1}$ was associated with a greater inhibition of efficacy of PDT.

Local administration of noradrenaline ($200\,\mu l$ of $0.02\,mg\,ml^{-1}$, amounting to $0.2\,mg\,kg^{-1}$ into tumours concurrently with the administration of HPD ($50\,mg\,kg^{-1}$ i.p.) 2h before irradiation, also greatly inhibited the response to PDT. Eight out of 10 tumours were still palpable 24h after irradiation ($TC_{50}$ of $<1$ day compared to 8 days). HPD fluorescence in the tumours was also greatly inhibited by local infiltration of noradrenaline. Controls of local noradrenaline alone or noradrenaline plus light had no macroscopic effect on the tumours. These results would indicate that the effect of noradrenaline on uptake of HPD into tumours could be at least partly due to effects of noradrenaline on the tumour blood vessels and not merely due to systemic vasoconstriction affecting the distribution or circulation of HPD in the mouse.

Under the conditions tested, the alpha blocker phenoxybenzamine did not prevent the inhibition of PDT by noradrenaline, an alpha agonist (Figure 2). Indeed the inhibition of PDT was enhanced, most tumours being macroscopically unaffected. Phenoxybenzamine itself administered concurrently with HPD 2h before irradiation, also inhibited PDT (Figure 2). The inhibition of PDT by noradrenaline was not altered by concurrent administration of the beta blocker propranolol (Figure 2). Higher doses of propranolol in combination with noradrenaline were fatal to the mice. All drugs alone, drugs plus HPD (without irradiation) and drugs plus irradiation (without HPD) had no effect on the rate of tumour growth.

*Calcium channel blockers*

Verapamil ($2\,mg\,kg^{-1}$) potentiated PDT when administered concurrently with HPD 24h before irradiation (Figure 3). No greater effect was seen using this drug at $10\,mg\,kg^{-1}$. A marked increase in fluorescence in frozen sections 24h after injection of verapamil ($10\,mg\,kg^{-1}$) plus HPD suggested that verapamil increased uptake or retention of HPD in the tumours. A second calcium channel blocking agent, nifedepine, was also tested. Nifedepine, being extremely insoluble in aqueous solutions, was dissolved in 5% dimethyl sulphoxide (DMSO) in saline and then injected concurrently with HPD. Both the photodynamic effect and the uptake of HPD, as assessed by fluorescence, were greatly inhibited. However, 5% DMSO without nifedepine administered with HPD also inhibited both the uptake of HPD and the photodynamic response. The reason for this inhibition is unclear. Thus the influence of nifedepine on PDT could not be assessed. Diltiazem was also tested (Figure 3). In contrast to verapamil, when diltiazem ($10\,mg\,kg^{-1}$) was administered concurrently with HPD 24h before irradiation, there was no change in the $TC_{50}$, which remained at 4 days. The fluorescence of HPD in tumours was similarly unaffected by diltiazem. Controls of drugs alone, drug plus HPD or drug plus light had no effect on the tumours.

Verapamil ($2\,mg\,kg^{-1}$) administered 24 and 48h after irradiation significantly increased the $TC_{50}$ from 3.6 to 6 days. However, in contrast, neither nifedepine ($2\,mg\,kg^{-1}$, dissolved in 5% DMSO in saline) or diltiazem (10 or $50\,mg\,kg^{-1}$) had any effect on the $TC_{50}$ when administered 24 and 48h after PDT (Figure 4). The insolubility of nifedepine in aqueous solutions limited the doses at which it could be tested. A control solution of 5% DMSO in saline administered 24 and 48h after PDT had no effect on the rate of recurrence of the tumours. Verapamil ($10\,mg\,kg^{-1}$) administered to mice at the time of tumour transplant also had no effect on the rate of tumour growth. The mean time for tumours to become palpable was $7.8 \pm 1.4$ days when verapamil was administered with transplants and $7.5 \pm 1.3$ days without verapamil.

**Figure 3** Influence of calcium channel blocking drugs administered concurrently with HPD. Mice with Lewis lung carcinoma were given HPD and calcium channel blocking drugs and the tumours irradiated 24h later with $225\,J\,cm^{-2}$ red light. Each bar represents the mean $\pm$ s.d. of two experiments. A, HPD ($30\,mg\,kg^{-1}$ i.p.); B, HPD plus verapamil (Isoptin, Knoll AG, $2\,mg\,kg^{-1}$ i.p.); C, HPD plus verapamil ($2\,mg\,kg^{-1}$ i.v.); D, HPD plus verapamil ($10\,mg\,kg^{-1}$ i.p.); E, HPD plus diltiazem (ICI Australia, $10\,mg\,kg^{-1}$ i.p.). $^{*}P<0.02$; $^{**}P<0.01$.

*Influence of verapamil on the photoactivity of HPD* in vitro

Verapamil could act on PDT by affecting the supply of HPD to tumours, or by a direct effect on tumour cells. In an attempt to distinguish between these possibilities, the influence of verapamil on photodynamic destruction of tumour cells was examined *in vitro*. Verapamil had no effect on $^{51}Cr$ release from HPD-sensitised Lewis lung carcinoma cells (Figure 5). Uptake of HPD into Lewis lung carcinoma cells was assessed by flow cytometry. The mean fluorescence was proportional to the concentration of HPD added to the incubation mixture (data not shown), suggesting mean fluorescence was a measure of intracellular HPD concentration. Verapamil did not markedly affect the mean fluorescence of Lewis lung carcinoma cells incubated in HPD (Figure 6), suggesting HPD uptake was not affected by verapamil. Neither verapamil ($10$–$50\,\mu g\,ml^{-1}$) alone nor verapamil plus light increased $^{51}Cr$ release above background. Since incubation of cells in verapamil alone at a concentration of $100\,\mu g\,ml^{-1}$ caused some release of $^{51}Cr$ above background, higher concentrations of verapamil were not tested.

**Figure 4** Influence of calcium channel blocking drugs administered 24 and 48 h after PDT. Mice with Lewis lung carcinoma were given HPD (30 mg kg$^{-1}$ i.p.) and tumours irradiated 24 h later with 225 J cm$^{-2}$ red light. The calcium channel blocking drugs were administered i.p. 24 and 48 h after irradiation. Each bar represents the mean ± s.d. of two experiments. A, HPD and light only; B, verapamil (2 mg kg$^{-1}$); C, nifedepine (2 mg kg$^{-1}$); D, diltiazem (10 mg kg$^{-1}$); E, diltiazem (50 mg kg$^{-1}$). *$P$<0.02.

**Figure 5** Influence of verapamil on PDT *in vitro*. $^{51}$Cr-labelled Lewis lung carcinoma cells were incubated for 1 h at 37°C with HPD (25 μg ml$^{-1}$) and verapamil (1–100 μg ml$^{-1}$) and irradiated for 0–20 min. Percentage $^{51}$Cr was determined immediately after irradiation. Each point represents the mean ± s.d. of triplicates. ●, HPD (25 μg ml$^{-1}$); ○, HPD plus verapamil (10 μg ml$^{-1}$); ■, HPD plus verapamil (25 μg ml$^{-1}$); □, HPD plus verapamil (50 μg ml$^{-1}$).

**Figure 6** Effect of verapamil on uptake of HPD *in vitro*. Lewis lung carcinoma cells were incubated for 1 h at 37°C with HPD (0 or 25 μg ml$^{-1}$) and verapamil (0–100 μg ml$^{-1}$), washed and suspended in PBS. Mean fluorescence intensity was read on a Becton Dickinson FACS analyser. Each point represents the mean ± s.d. of triplicates. ●, no HPD; ○, HPD (25 μg ml$^{-1}$).

### Discussion

The influence on PDT of drugs affecting vasculature was examined using a transplantable tumour model in mice. Noradrenaline, propranolol and hydralazine inhibited PDT if given 2 h before tumours were irradiated, but had no effect when administered with HPD 24 h before irradiation. The fluorescence of HPD in tumours was reduced 2 h after administration of noradrenaline with HPD, but not after 24 h. Phenoxybenzamine, an alpha receptor blocking agent and the beta blocker propranolol did not block the inhibitory effect of noradrenaline on PDT. Noradrenaline acts mainly on alpha receptors (Weiner, 1985). Administration of noradrenaline, hydralazine or propanolol immediately before irradiation had no effect on the $TC_{50}$.

There is controversy as to whether blood vessels of tumours respond to vasoactive drugs. Blood flow in rat tumours was reduced by local administration of noradrenaline (Mattsson *et al.*, 1980) and vasoconstriction was observed directly by microangiography (Mattsson *et al.*, 1981). Decreasing vascular responses to propranolol, papaverine and dihydralazine have been observed as tumours age (Wickersham *et al.*, 1977; Peterson & Mattsson, 1984). As tumours enlarge, the blood vessels become stretched and tortuous, lose adrenergic innervation and show a relative lack of smooth muscle (Mattsson & Peterson, 1981). The inhibition of PDT by vasolidators administered 2 h before irradiation could be explained by dilatation of normal vessels causing diversion of blood from tumours. Reduction of tumour blood flow has been demonstrated after the intravenous doses of noradrenaline used in this study and this effect was blocked by phenoxybenzamine (Mattsson *et al.*, 1978). However, since phenoxybenzamine itself inhibited PDT, this drug could not be used to block the inhibitory action of noradrenaline on PDT. Administration of these drugs immediately before irradiation had no effect on the response of the tumours to PDT, suggesting that the degree of vasodilation or vasoconstriction of the tumour vasculature at the time of irradiation does not influence the outcome of PDT.

Damage to tumours by PDT appears to be mediated by toxic oxygen species, particularly singlet oxygen and hydroxyl radicals (Das *et al.*, 1985). It is possible that vasoactive drugs alter tumour responses to PDT by altering the oxygen tension in the tumours and hence altering the production of toxic oxygen species. Vasoactive drugs have been reported to alter oxygen tension (Kruuv *et al.*, 1967).

Verapamil, a calcium channel blocking agent and vasodilator, enhanced the efficacy of PDT. Enhancement of HPD fluorescence in tumours suggested that this resulted from increased uptake of HPD. However, since vasodilators inhibited both uptake of HPD and the degree of photodynamic damage, an effect of verapamil other than its vasodilatory action is probably responsible. The capacity of verapamil to affect blood flow of tumours is also controversial. In one study, tumour blood flow was increased by verapamil without altering blood flow in normal tissues (Kaelin *et al.*, 1982), but no enhancement of tumour blood flow was detected in another study (Robinson *et al.*, 1985). It is of interest that verapamil increases the number of low density lipoprotein (LDL) receptors on the surface of cells (Stein *et al.*, 1985), as it has been suggested that HPD may be taken up into cells by LDL receptors (Candide *et al.*, 1986).

Verapamil enhances the effect of cytotoxic drugs in other systems. Tumour cell lines, including Lewis lung carcinoma used in this study, resistant to adriamycin and vincristine, were made sensitive by verapamil (Tsuruo *et al.*, 1982, 1983*a, b, c*). Intracellular concentrations of cytotoxic drugs were elevated, suggesting that verapamil enhanced uptake or inhibited transport of drugs out of cells. Our studies *in vitro* did not indicate a direct action of verapamil on the tumour cells. When Lewis lung carcinoma cells were incubated in HPD plus verapamil, HPD fluorescence and $^{51}Cr$ release were not increased significantly.

The most important effect of verapamil in enhancing PDT appears not to be the enhancement of uptake of HPD into tumours, but prolongation of the tumour-free interval when the drug is administered after irradiation. At least two mechanisms may be suggested to explain this phenomenon: (1) enhancement of the effect of hypoxia induced by capillary damage after PDT and (2) inhibition of regrowth of the tumour from surviving cells. Since virtually all non-malignant cells appear to proliferate and differentiate in response to appropriate growth factors, it seems likely that capillaries and host stromal cells may only grow in association with multiplying tumour cells if they also receive the necessary growth factors. Verapamil could inhibit some steps necessary for this process. High-dose cortisone, in combination with heparin, has been shown to inhibit angiogenesis (Folkman *et al.*, 1983; Crum *et al.*, 1985). Glucocorticoids administered after PDT also inhibit recurrence of tumours (Cowled *et al.*, 1985*a*). It is also of interest that the effects on PDT of verapamil are not common to all calcium channel blocking drugs and the reasons for these differences are not known. Diltiazem, a drug with pharmacological action similar to that of verapamil (Needleman *et al.*, 1985), had no effect on the uptake of HPD. Diltiazem and nifedepine also had no effect on the rate of recurrence of tumours after PDT.

We conclude that many vasoactive drugs may modify the uptake of HPD, inhibiting PDT if tumours are irradiated shortly afterwards. Verapamil acts differently, both enhancing the uptake of HPD and acting after photodynamic destruction to inhibit the regrowth of tumours. Verapamil may be very useful clinically to enhance the efficacy of PDT and experimentally to study the regrowth of tumours after PDT.

We would like to thank Lorraine Mackenzie and Sharon Bransbury for expert technical assistance and Joseph Webster, Department of Clinical Immunology, Flinders Medical Centre for performing the flow cytometry assays. Nifedipine was kindly supplied by Bayer Australia Limited and diltiazem by ICI Australia Operations Proprietary Limited.

### References

BENSON, R.C. (1985). Treatment of diffuse transitional cell carcinoma *in situ* by whole bladder hematoporphyrin derivative photodynamic therapy. *J. Urol.*, **134,** 675.

BERENBAUM, M.C., HALL, G.W. & HOYES, A.D. (1986). Cerebral photosensitisation by haematoporphyrin derivative. Evidence for an endothelial site of action. *Br. J. Cancer*, **53,** 81.

BUGELSKI, P.J., PORTER, C.W. & DOUGHERTY, T.J. (1981). Autoradiographic distribution of hematoporphyrin derivative in normal and tumor tissue of the mouse. *Cancer Res.*, **41,** 4606.

CANDIDE, C., MORLIERE, P., MAZIERE, J.C. and 5 others (1986). *In vitro* interaction of the photoactive anticancer porphyrin derivative photofrin II with low density lipoprotein, and its delivery to cultured human fibroblasts. *FEBS Lett.*, **207,** 133.

COWLED, P.A. (1986). Photodynamic therapy with haematoporphyrin derivative. Thesis submitted to the University of Adelaide for the Degree of Doctor of Philosophy.

COWLED, P.A. & FORBES, I.J. (1985). Photocytotoxicity *in vivo* of haematoporphyrin derivative components. *Cancer Lett.*, **28,** 111.

COWLED, P.A., MACKENZIE, L. & FORBES, I.J. (1985*a*). Potentiation of photodynamic therapy with haematoporphyrin derivative by glucocorticoids. *Cancer Lett.*, **29,** 107.

COWLED, P.A., FORBES, I.J., SWINCER, A.G., TRENERRY, V.C. & WARD, A.D. (1985*b*). Separation and phototoxicity *in vitro* of some of the components of haematoporphyrin derivative. *Photochem. Photobiol.*, **41,** 445.

CRUM, R., SZABO, S. & FOLKMAN, J. (1985). A new class of steroids inhibits angiogenesis in the presence of heparin or a heparin fragment. *Science*, **230,** 1375.

DAS, M., DIXIT, R., MUKHTAR, H. & BICKERS, D.R. (1985). Role of active oxygen species in the photodestruction of microsomal cytochrome P-450 and associated monooxygenases by haematoporphyrin derivative in rats. *Cancer Res.*, **45,** 608.

DOUGHERTY, T.J., BOYLE, D.G., WEISHAUPT, K.R. and 4 others (1983). Photoradiation therapy: clinical and drug advances. In *Advances in Experimental Medicine and Biology, vol. 160: Porphyrin Photosensitization*, Kessel, D. & Dougherty, T.J. (eds) p. 3. Plenum Press: New York.

FOLKMAN, J., LANGER, R., LINHARDT, R.J., HAUDENSCHILD, D. & TAYLOR, S. (1983). Angiogenesis inhibition and tumor regression caused by heparin or a heparin fragment in the presence of cortisone. *Science*, **221,** 719.

FORBES, I.J., COWLED, P.A., LEONG, A.S.-Y. and 4 others (1980). Phototherapy of human tumours using haematoporphyrin derivative. *Med. J. Aust.*, **2,** 489.

HAYATA, Y., KATO, H., KONAKA, C. and 6 others (1984). Photoradiation therapy with hematoporphyrin derivative in early and stage I lung cancer. *Chest*, **86,** 169.

HENDERSON, B.W., WALDOW, S.M., MANG, T.S., POTTER, W.R., MALONE, P.B. & DOUGHERTY, T.J. (1985). Tumour destruction and kinetics of tumor cell death in two experimental mouse tumors following photodynamic therapy. *Cancer Res.*, **45,** 572.

JACKA, F. & BLAKE, A.J. (1983). A lamp for cancer phototherapy. *Aust. J. Phys.*, **36,** 221.

KAELIN, W.G., SHRIVASTAV, S., SHAND, D.G. & JIRTLE, R.L. (1982). Effect of verapamil on malignant tissue blood flow in SMT-2A tumor-bearing rats. *Cancer Res.*, **42,** 3944.

KRUUV, J.A., INCH, W.R. & McCREDIE, J.A. (1967). Blood flow and oxygenation of tumours in mice. *Cancer*, **20,** 60.

LEE SEE, K., FORBES, I.J. & BETTS, W.H. (1984). Oxygen dependency of photocytotoxicity with haematoporphyrin derivative. *Photochem. Photobiol.*, **39,** 631.

MATTSSON, J., ALPSTEN, M., APPELGREN, L. & PETERSON, H.-I. (1980). Influence of noradrenaline on local tumour blood flow. *Eur. J. Cancer*, **16,** 99.

MATTSSON, J., APPELGREN, L., KARLSSON, K. & PETERSON, H.-I. (1978). Influence of vasoactive drugs and ischaemia on intra-tumour blood flow distribution. *Eur. J. Cancer*, **14**, 761.

MATTSSON, J., APPELGREN, K.L., KARLSSON, L. & PETERSON, H.-I. (1981). Influence of vasoactive drugs on local tumor blood flow. *Bibl. Anat.*, **20**, 614.

MATTSON, J. & PETERSON, H.-I. (1981). Influence of vasoactive drugs on tumour blood flow (review). *Anticancer Res.*, **1**, 59.

MUSSER, D.A. & DATTA-GUPTA, N. (1984). Inability to elicit rapid cytocidal effects on L1210 cells derived from porphyrin-injected mice following *in vitro* photoirradiation. *J. Natl Cancer Inst.*, **72**, 427.

NEEDLEMAN, P., CORR, P.B., & JOHNSON, E.M. (1985). Drugs used for the treatment of angina: organic nitrates, calcium channel blockers, and beta-adrenergic antagonists. In *Goodman and Gilman's the Pharmacological Basis of Therapeutics*, 7th edn, Gilman, A.G., Goodman, L.S., Rall, T.W. & Murad, F. (eds) p. 806. Macmillan: New York.

PETERSON, H.-I. & MATTSSON, J. (1984). Vasoactive drugs and tumor blood flow. *Biorheology*, **21**, 503.

ROBINSON, B.A., CLUTTERBUCK, R.D., MILLAR, J.L. & MCELWAIN, T.J. (1985). Verapamil potentiation of melphalan cytotoxicity and cellular uptake in murine fibrosarcoma and bone marrow. *Br. J. Cancer*, **52**, 813.

SELMAN, S.H., KREIMER-BIRNBAUM, M., KLAUNIG, J.E., GOLDBLATT, P.J., KECK, R.W. & BRITTON, S.L. (1984). Blood flow in transplantable bladder tumors treated with hemato-porphyrin derivative and light. *Cancer Res.*, **44**, 1924.

SELMAN, S.H., MILLIGAN, A.J., KREIMER-BIRNBAUM, M., KECK, R.W., GOLDBLATT, P.J. & BRITTON, S.L. (1985). Hemato-porphyrin derivative photochemotherapy of experimental bladder tumors. *J. Urol.*, **133**, 330.

STAR, W.M., MARIJNISSEN, J.P.A., VAN DEN BERG-BLOK, A.E. & REINHOLD, H.S. (1984). Destructive effect of photoradiation on the microcirculation of a rat mammary tumor growing in 'sandwich' observation chambers. In *Porphyrin Localization and Treatment of Tumors*, Doiron, D.R. & Gomer, C.J. (eds) p. 637. Alan R. Liss: New York.

STEIN, O., LEITERSDORF, E. & STEIN, Y. (1985). Verapamil enhances receptor-mediated endocytosis of low-density lipoproteins by aortic cells in culture. *Arteriosclerosis*, **5**, 35.

TSURUO, T., IIDA, H., TSUKAGOSHI, S. & SAKURAI, Y. (1982). Increased accumulation of vincristine and adriamycin in drug-resistant P388 tumor cells following incubation with calcium antagonists and calmodulin inhibitors. *Cancer Res.*, **42**, 4730.

TSURUO, T., IIDA, H., NAGANUMA, K., TSUKAGOSHI, S. & SAKURAI, Y. (1983*a*). Promotion by verapamil of vincristine responsiveness in tumor cell lines inherently resistant to the drug. *Cancer Res.*, **43**, 808.

TSURUO, T., IIDA, H., NOJIRI, M., TSUKAGOSHI, S., & SAKURAI, Y. (1983*b*): Circumvention of vincristine and adriamycin resistance *in vitro* and *in vivo* by calcium influx blockers. *Cancer Res.*, **43**, 2905.

TSURUO, T., IIDA, H., TSUKAGOSHI, S., SAKURAI, Y. (1983*c*). Potentiation of vincristine and adriamycin effects in human hemopoietic tumor cell lines by calcium antagonists and calmodulin inhibitors. *Cancer Res.*, **43**, 2267.

WEINER, N. (1985). Norepinephrine, ephinephrine, and the sympathomimetic amines. In *Goodman and Gilman's the Pharmacological Basis of Therapeutics*, 7th edn, Gilman, A.G., Goodman, L.S., Rall, T.W. & Murad, F. (eds) p. 145. Macmillan: New York.

WICKERSHAM, J.K., BARRETT, W.P., FURUKAWA, S.B., PUFFER, H.W. & WARNER, N.E. (1977). An evaluation of the response of the microvasculature in tumors in C3H mice to vasoactive drugs. *Bibl. Anat.*, **15**, 291.